# Non‐attendance at outpatient clinic appointments by children with cerebral palsy

**DOI:** 10.1111/dmcn.15197

**Published:** 2022-03-04

**Authors:** Simon P. Paget, Sarah McIntyre, Shona Goldsmith, Katarina Ostojic, Jane Shrapnel, Francisco Schneuer, Mary‐Clare Waugh, Maria Kyriagis, Natasha Nassar

**Affiliations:** ^1^ Faculty of Medicine and Health The Children’s Hospital at Westmead Clinical School University of Sydney Sydney New South Wales Australia; ^2^ The Children’s Hospital at Westmead Westmead New South Wales Australia; ^3^ Specialty of Child & Adolescent Health Sydney Medical School Faculty of Medicine & Health Cerebral Palsy Alliance Research Institute The University of Sydney Sydney New South Wales Australia; ^4^ Sydney Children’s Hospital Randwick New South Wales Australia

## Abstract

**Aim:**

To determine factors that influence non‐attendance at outpatient clinics by children with cerebral palsy (CP).

**Method:**

This was a retrospective cohort study of 1395 children with CP (59.6% male; born 2005 to 2017) identified from the New South Wales (NSW)/Australian Capital Territory CP Register, who had scheduled appointments at outpatient clinics at two NSW tertiary paediatric hospitals between 2012 and 2019. Associations between sociodemographic, clinical, and process‐of‐care factors and non‐attendance were examined using multivariate logistic regression with generalized estimating equations.

**Results:**

A total of 5773 (12%) of 50 121 scheduled outpatient days were not attended. Non‐attendance increased over time (average increase 5.6% per year, 95% confidence interval [CI]: 3.7–7.3). Older children aged 5 to 9 years (adjusted odds ratio [aOR] 1.11; 95% CI: 1.02–1.22) and 10 to 14 years (aOR 1.17; 95% CI: 1.03–1.34), socioeconomic disadvantage (aOR 1.29; 95% CI: 1.11–1.50), previous non‐attendance (aOR 1.38; 95% CI: 1.23–1.53), and recent rescheduled or cancelled appointments (aOR 1.08; 95% CI: 1.01–1.16) were associated with increased likelihood of non‐attendance.

**Interpretation:**

One in eight outpatient appointments for children with CP were not attended. Non‐attendance was associated with increasing age, socioeconomic disadvantage, previous non‐attendance, and recent rescheduled or cancelled appointments. Identifying specific barriers and interventions to improve access to outpatient services for these groups is needed.

**What this paper adds:**

- Twelve per cent of scheduled appointments for children with cerebral palsy are not attended.
- Proportions of appointments not attended has increased over the last decade.
- Increasing age and socioeconomic disadvantage increase the likelihood of non‐attendance.
- Previous non‐attendance and recent cancelled or rescheduled appointments increase the likelihood of further non‐attendance.

## What this paper adds

This study determines factors that influence non‐attendance at outpatient clinics by children with cerebral palsy (CP). One in eight outpatient appointments for children with CP were not attended. Non‐attendance was associated with increasing age, socioeconomic disadvantage, previous non‐attendance, and recent rescheduled or cancelled appointments.

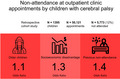

**Abbreviations**
ACT: Australian Capital Territory
NAP: Non‐admitted patient
NSW: New South Wales
SCHN: Sydney Children’s Hospitals Network

Cerebral palsy (CP) is a neurodevelopmental condition characterized by a permanent disorder of movement and posture attributed to non‐progressive disturbances in early brain development.^1^ CP is the most common cause of physical disability in childhood, with a birth prevalence of approximately 2.0 per 1000 live births in most high‐income countries,^2^ although prevalence has declined in recent birth years in Australia.^3^ For many children with CP, the motor disorder is accompanied by neurological disorders (e.g. epilepsy), diseases of other body systems (e.g. respiratory, digestive system),^4^ and musculoskeletal deformities^5^, ^6^ (e.g. scoliosis, hip displacement) that further complicate their health.

The long‐term management of CP and its associated health conditions and complications is conducted by health services,^7^ with most management occurring in outpatient settings. As children with CP often have complex health needs, specialty medical and surgical outpatient services are generally centralized in children’s hospitals and frequently involve multidisciplinary teams including medical, nursing, and allied health professionals.

Non‐attendance at scheduled outpatient appointments is recognized as a major issue across the health care system and health conditions. At a patient level, non‐attendance may represent a missed opportunity for early diagnosis of a health‐related problem, or the initiation of an intervention to improve the health or well‐being of their child. In children with neurological conditions, it may result in increased, unplanned health care use such as emergency department presentations.^8^ At a health service level, non‐attendance is recognized to increase health care costs, decrease services’ effective capacity, and add to waiting times for consultations and procedures.^9^

In this context, understanding factors associated with non‐attendance at outpatient appointments is important to enable the identification of strategies to improve attendance and health outcomes for children with CP.^10^ A systematic review of non‐attendance across patient groups identified multiple factors that are likely to be relevant, including elements related to the individual (younger age, lower socioeconomic status, history of previous non‐attendance) and those related to the clinic (e.g. specialty type) and service.^9^ Given the complexity and diversity of CP, other aspects, such as the severity of CP and comorbidities, may also be important. We aimed to explore the factors associated with non‐attendance by children with CP at specialty outpatient clinics located at two locations across a tertiary children’s hospital network.

## METHOD

### Study population and data sources

We conducted a retrospective cohort study of children with CP, born from 2005 to 2017, who attended outpatient clinics at two children’s hospitals in New South Wales (NSW) that provide services for children in NSW and the Australian Capital Territory (ACT). Children with CP were identified from the NSW/ACT CP Register (*n*=1764), a population‐based database with multiple ascertainment strategies. The Register contains details of individuals with CP who were born or live in NSW or the ACT, including demographic and clinical (motor type, severity of CP, presence of comorbidities) information. For each child, corresponding information was ascertained on outpatient appointments scheduled at either of two tertiary paediatric hospitals in metropolitan Sydney: Sydney Children’s Hospital, Randwick and the Children’s Hospital at Westmead (as part of the Sydney Children’s Hospitals Network [SCHN]) between 1st January 2012 and 31st December 2019. This time frame was chosen because data before 2012 were incomplete due to changes in the data collection processes. Outpatient data were obtained from the SCHN non‐admitted patient (NAP) administrative data collection. SCHN NAP data is based on two sources: data documenting scheduled outpatient appointments and patient‐level clinician activity including demographic information, clinical specialty type, location, attendance/non‐attendance, and clinician discipline. The accuracy of the NAP data collection is ensured as it is a statutory with the NSW Ministry of Health mandating the collection and reporting of patient level non‐admitted activity for all clinical and/or therapeutic services provided or contracted by NSW Health. Outpatient clinics at both hospitals are provided under a government universally funded system (either state‐funded or Medicare) without a fee to the patient, typically 8am to 5pm, Monday to Friday.

### Study outcomes

The main study outcome was frequency of scheduled outpatient appointments categorized as attended or not attended. The data available in the SCHN NAP did not discriminate between appointments rescheduled or cancelled by the hospital for administrative reasons (e.g. staff being unavailable) and those rescheduled by families. Scheduled outpatient appointments were categorized based on clinical specialty (see Table S1) and health care professionals seen were categorized by discipline (medical/dental, nursing, allied health, other). To adjust for varied scheduling practices (e.g. some specialties scheduled multiple appointments with health care professionals of different disciplines on the same day), scheduled outpatient appointments were converted to outpatient days. At each (attended) outpatient day, a child could be reviewed by different clinical specialties and seen by multiple health care professionals of different disciplines. A flow diagram presenting an overview of the study processes including study exclusions is shown in Figure S1.

### Patient sociodemographic, clinical, and process of care factors

Patient sociodemographic and clinical factors were collected from the CP Register and SCHN NAP, and included demographic information on age at appointment, sex, preferred language, and country of birth. Patients’ postcode of residence was used to estimate socioeconomic disadvantage and geographical remoteness. Socioeconomic disadvantage was measured with reference to the general population, using the Index of Relative Socioeconomic Disadvantage and categorizing into quintiles (quintile 1 being the most disadvantaged and quintile 5 being the least disadvantaged).^11^ Geographical remoteness was defined using the Australian Statistical Geography Standard, which categorizes populated localities as major cities, inner/outer regional, and rural/remote areas) based on ease of access to services via road network.^12^ Clinical variables included Gross Motor Function Classification System (GMFCS) classification (dichotomized into levels I–III [ambulant] and IV–V [non‐ambulant]),^13^ predominant motor type (grouped into spastic, dyskinetic, and other [ataxia, hypotonia, those identified as ‘early and at risk’ of CP]), and the presence of comorbidities of epilepsy and intellectual disability (dichotomized as ‘yes’ or ‘no’).

Process‐of‐care factors were identified using NAP data. Recent multidisciplinary team care was defined as review by two or more health care professionals from different disciplines at the previous outpatient day (visit). Recent experience of care coordination was defined as review by two or more different clinical specialties at the previous outpatient day. Recent non‐attendance was defined as non‐attendance at the previous outpatient day. Appointments that were rescheduled or cancelled were also identified. Recent rescheduled or cancelled appointments were defined as one or more rescheduled/cancelled appointment in the previous 6 months. Where there was no previous recorded appointment (e.g. at the first scheduled outpatient day during the study period), these process‐of‐care factors were classified as ‘no’.

### Statistical analysis

Children who were scheduled to attend clinic appointments at either of the two hospitals on at least one occasion (*n*=1395) were described in terms of their demographic and clinical features. Proportions, counts, and rates of scheduled appointments by specialty type were compared. Characteristics of children reviewed by major specialties and proportions of scheduled outpatient appointments by age group were compared using χ^2^ tests. Associations between patient factors and non‐attendance were assessed using univariate and multivariate logistic regression including date of appointment, child sociodemographic and clinical factors, and process‐of‐care measures. Multivariate analyses were conducted using generalized estimating equations and an exchangeable correlation structure to account for repeated outpatient attendances by the same child. Analyses were conducted using SAS 9.4 (SAS Institute, Cary, NC, USA). The study was approved by the SCHN human research ethics committee (2019/ETH11829).

## RESULTS

We identified 1395 children from the NSW/ACT CP Register who had one or more outpatient appointment scheduled during the study period (Table 1). Of these children, 831 (59.6%) were male; most (1340; 96.1%) lived either in major cities or inner regional areas.

**TABLE 1:** Characteristics of 1395 children with cerebral palsy with scheduled outpatient appointments, 2012 to 2019

Demographic/clinical factor	*n* (%)
Sex	
Male	831 (59.6)
Female	564 (40.4)
Country of birth	
Australia	1298 (93.7)
Overseas	88 (6.3)
Preferred language	
English	1214 (91)
Other	120 (9)
Remoteness	
Major cities of Australia	941 (67.7)
Inner regional Australia	399 (28.7)
Outer regional Australia	42 (3)
Remote Australia	7 (0.5)
State/territory of residence	
New South Wales	1323 (95.9)
Australian Capital Territory	57 (4.1)
IRSD quintile	
1 (most disadvantaged)	274 (19.7)
2	210 (15.1)
3	275 (19.8)
4	288 (20.7)
5 (least disadvantaged)	342 (24.6)
GMFCS level	
I–III	998 (74.5)
IV–V	342 (25.5)
Predominant motor type	
Spastic	1010 (73.6)
Dyskinetic	192 (14.0)
Other	171 (12.5)
Intellectual disability	
Yes	645 (46.2)
No	515 (36.9)
Not reported	235 (16.8)
Epilepsy	
Yes	394 (28.2)
None or resolved	796 (57.1)
Not reported	205 (14.7)

Abbreviations: GMFCS, Gross Motor Function Classification System; IRSD, Index of Relative Socioeconomic Disadvantage.

There was a total of 50 121 scheduled outpatient days during 2012 to 2019; each child had a median of 4.8 (interquartile range [IQR)] 2.0–7.9) scheduled appointments per year. There was variation in the frequency and involvement of different specialties (Table 2). Most children were reviewed one or more times by rehabilitation medicine (82.2%), allied health (78.9%), and neurology/neurosurgery (55.6%) clinics. These clinics were also the most frequently attended (Table 2). There were differences between the groups of children reviewed by different specialties (Table S2). Compared with children without each respective comorbidity, children with epilepsy (odds ratio [OR] 6.28; 95% confidence interval [CI]: 4.68–8.44) and intellectual disability (OR 3.06; 95% CI: 2.41–3.90) were substantially more likely to be seen in neurology/neurosurgery clinics; children with non‐ambulant CP were more likely to be reviewed in orthopaedic clinics (OR 3.69; 95% CI: 2.84–4.79) (Table S2). There were also differences in specialty scheduled outpatient days between age groups (Table S3). The 0 to 4‐year age group attended 54% of neurology outpatient days (compared with 40% of total outpatient days) and the 10 to 14‐year age group attended 11% of neurology outpatient days (compared with 15% of total outpatient days). In contrast, the 0 to 4‐year age group attended 18% of orthopaedic outpatient days while the 10 to 14‐year age group attended 30% of orthopaedic outpatient days.

**TABLE 2:** Number and proportion of children with cerebral palsy attending scheduled outpatient appointments and non‐attendance by specialty group, 2012 to 2019

Specialty group	Children attending outpatient clinics *n* (%)	Frequency of outpatient days *n* (%)	Number of scheduled appointments/year mean (SD)	Scheduled appointments not attended *n* (%)
Allied health	1100 (78.9)	19 008 (37.9)	2.2 (2.7)	2187 (11.5)
General medicine	297 (21.3)	1863 (3.7)	0.2 (0.9)	416 (22.3)
Rehabilitation medicine	1147 (82.2)	14 918 (29.8)	1.8 (2.1)	1651 (11.1)
Neurology/neurosurgery	776 (55.6)	4982 (9.9)	0.7 (1.6)	469 (9.4)
Other medical specialty	776 (55.6)	6172 (12.3)	0.8 (2)	669 (10.8)
General surgery	305 (21.9)	1030 (2.1)	0.1 (0.5)	127 (12.3)
Orthopaedics	604 (43.3)	4433 (8.8)	0.5 (0.9)	655 (14.8)
Other surgical specialty	681 (48.8)	4559 (9.1)	0.6 (1)	604 (13.2)
Medical imaging	625 (44.8)	4620 (9.2)	0.6 (1.2)	214 (4.6)
Total	1395 (100)	50 121 (100)	6.1 (6.1)	5773 (11.5)

Numbers and percentages do not sum to totals as children may attend multiple specialty clinics.

**FIGURE 1:**
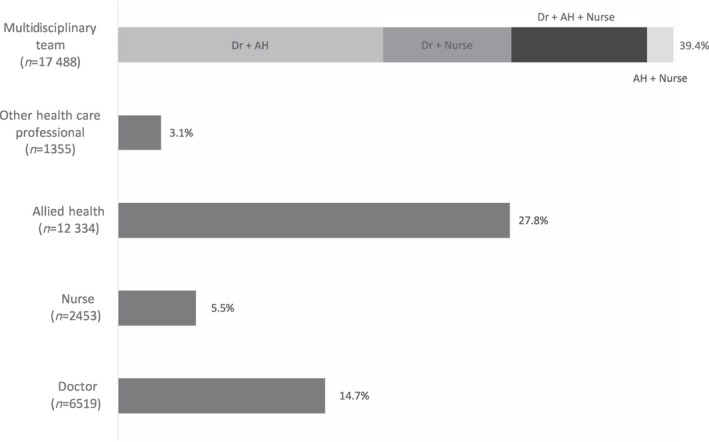
Proportion of outpatient days attended by children with cerebral palsy (by health care professional discipline). The health care professional involved was unknown in 4199 (9.5%) outpatient days; all groups are mutually exclusive. AH, allied health

Most (*n*=44 348, 88.5%) scheduled outpatient days were attended, with children seen by a single provider in about half (51.1%) of all attended outpatient days (Fig. 1). Multidisciplinary team care was provided in 39.4% of attended outpatient days and most involved a doctor and an allied health professional (47.7%), doctor and nurse (23.1%), or doctor, nurse, and allied health professional (24.4%) (Fig. 1). Care coordination of multiple specialty appointments occurred in 19.9% (*n*=8813) of all attended outpatient days.

**FIGURE 2:**
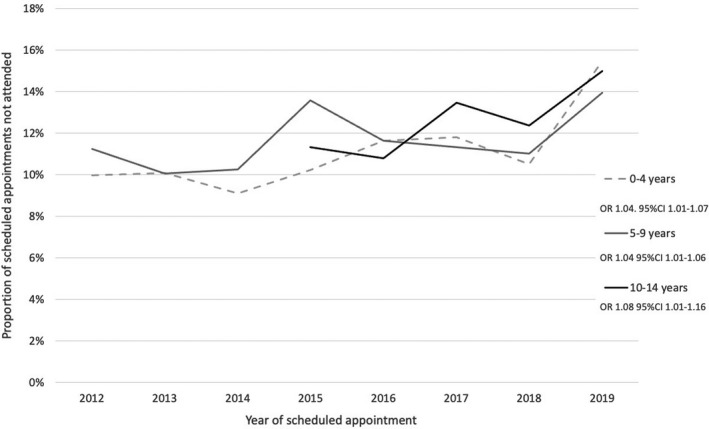
Proportion of scheduled outpatient clinic appointments not attended by year and age group in children with cerebral palsy at a children’s hospitals network

A total of 5773 (11.5%) scheduled outpatient days were not attended (Table 2). The rate of non‐attendance increased on average by 5% per year (OR 1.05; 95% CI: 1.04–1.07) from 11.5% in 2012 to 14.2% in 2019. The rate of increase was similar among the 0 to 4‐year age group (OR 1.04; 95% CI: 1.01–10.07), 5 to 9‐year age group (OR 1.04; 95% CI: 1.01–1.06), and 10 to 14‐year age group (OR 1.08; 95% CI: 1.01–1.16) (Fig. 2).

The association between sociodemographic, clinical, and process‐of‐care factors and non‐attendance is shown in Table 3. After adjusting for all factors, increased likelihood of non‐attendance was associated with older age children 5 to 9 years (aOR 1.11; 95% CI: 1.02–1.22) and 10 to 14 years (aOR 1.17; 95% CI: 1.03–1.34) and greater socioeconomic disadvantage (Index of Relative Socioeconomic Disadvantage quintile 1: aOR 1.29; 95% CI: 1.11–1.50 and Index of Relative Socioeconomic Disadvantage quintile 2: aOR 1.50; 95% CI: 1.29–1.76) (Table 3). There was no statistical evidence of an association between clinical variables, such as GMFCS level and predominant motor type, and likelihood of non‐attendance. Recent experience of multidisciplinary team or coordinated care was not associated with the likelihood of non‐attendance. However, children with recent rescheduled or cancelled appointments (aOR 1.08; 95% CI: 1.01–1.16) or previous non‐attendance (aOR 1.38; 95% CI: 1.23–1.53) had increased odds of non‐attendance.

**TABLE 3:** Association between sociodemographic, clinical, and process‐of‐care factors with non‐attendance at outpatient clinics for children with cerebral palsy

Sociodemographic, clinical, and process‐of‐care factors	Univariate OR (95% CI)	Multivariate OR (95% CI)
Sociodemographic factors		
Year of appointment	1.05 (1.04–1.07)	1.04 (1.02–1.06)
Sex		
Male	1.04 (0.94–1.15)	1.01 (0.92–1.12)
Female	Reference	Reference
Age		
0–4 years	Reference	Reference
5–9 years	1.22 (1.12–1.34)	1.12 (1.03–1.23)
10–14 years	1.45 (1.3–1.63)	1.19 (1.04–1.35)
Country of birth		
Australia	Reference	Reference
Overseas	1.11 (0.92–1.34)	1.06 (0.8–1.27)
Preferred language		
English	Reference	Reference
Other	1.08 (0.92–1.26)	0.98 (0.83–1.16)
IRSD quintile		
1 (most disadvantaged)	1.32 (1.13–1.53)	1.30 (1.12–1.52)
2	1.50 (1.28‐ 1.76)	1.52 (1.30–1.78)
3	1.20 (1.04–1.40)	1.20 (1.03–1.39)
4	1.12 (0.96–1.30)	1.13 (0.97–1.30)
5 (least disadvantaged)	Reference	Reference
Remoteness		
Major cities of Australia	Reference	Reference
Regional/remote	1.04 (0.92–1.16)	0.95 (0.85–1.05)
Clinical factors		
GMFCS level		
I–III	Reference	Reference
IV–V	1.11 (1.00–1.24)	1.08 (0.95–1.23)
Predominant motor type		
Spastic	Reference	Reference
Dystonic	0.94 (0.83–1.08)	0.92 (0.79–1.06)
Other	0.96 (0.82–1.12)	0.97 (0.82–1.14)
Intellectual disability		
Yes	1.12 (1.01–1.23)	1.09 (0.97–1.22)
No	Reference	Reference
Epilepsy		
Yes	0.99 (0.89–1.10)	0.92 (0.82–1.04)
No	Reference	Reference
Process‐of‐care factors		
Last appointment with multidisciplinary team care		
Yes	1.05 (0.99–1.12)	1.01 (0.94–1.07)
No	Reference	Reference
Last appointment with care coordination		
Yes	1.05 (0.97–1.14)	1.03 (0.95–1.12)
No	Reference	Reference
Last appointment not attended		
Yes	1.40 (1.26–1.56)	1.32 (1.17–1.48)
No	Reference	Reference
Recent cancelled or rescheduled appointment		
Yes	1.14 (1.06–1.22)	1.08 (1.01–1.16)
No	Reference	Reference

Abbreviations: CI, confidence interval; GMFCS, Gross Motor Function Classification System; IRSD, Index of Relative Socioeconomic Disadvantage; OR, odds ratio.

## DISCUSSION

Non‐attendance at outpatient clinics for children with CP is a little‐researched area. We found non‐attendance to be associated with four factors: increasing age, socioeconomic disadvantage, previous non‐attendance at an outpatient clinic, and recent cancellation or rescheduling of an appointment. Non‐attendance was not associated with area of residence, CP severity, nor the presence of major comorbidities. Non‐attendance was also not associated with recent multidisciplinary team or coordinated care. Rates of non‐attendance increased during the study period.

Outpatient clinics are the dominant model through which the health system provides support for the management of chronic health conditions. Non‐attendance at outpatient clinics can, therefore, have important consequences for children with CP. Not attending an outpatient clinic appointment means a child misses an opportunity to receive timely (and evidence‐based) health interventions and/or engage in health surveillance and education. This may result in their using unplanned heath care (e.g. emergency departments) to support their needs,^8^ which can contribute over time to worse health outcomes. Our results suggest that children at greater socioeconomic disadvantage, who are already known to have higher rates of CP severity, intellectual disability, and comorbidities,^14^ are also inequitably exposed to these risks. It is encouraging that patients of overseas birth and non‐English speaking backgrounds, or those from regional or remote areas were not associated with non‐attendance.

That non‐attendance increases with age also requires further investigation. While this may represent changing priorities as children grow older, greater need for young‐person engagement, or reduced perceived need, some health conditions associated with CP are known (for the most part) to only become apparent with increasing age. Examples of this include scoliosis,^5^ and cognitive (e.g. attention‐deficit/hyperactivity disorder), affective, and anxiety disorders, which are also known to be more prevalent in children and adolescents with CP than other children.^15^ Our results support this finding, for example the development of musculoskeletal problems indicated by increased use of orthopaedic services in older age groups. Care fragmentation among multiple specialties as children grow older and new priorities arise may also result in children missing important aspects of care that are not typically addressed by all specialties. Awareness of this issue and ensuring services are adapted to be sensitive to changing needs and age‐appropriate is important.

Our findings are largely consistent with the research in non‐attendance at outpatient clinics in children (with CP and other health conditions). The rate of non‐attendance that we report is similar to that reported in a recent study of children with neurological conditions,^8^ although the reported rate of non‐attendance can vary substantially depending on setting. Studies in paediatric settings have suggested that factors relating to both individuals (e.g. sociodemographic factors, ethnicity, insurance status) and systems (e.g. waiting times for appointments, administrative error) are associated with non‐attendance.^16^, ^17^ Studies of adults in outpatient^18^ and primary care settings^19^ have also identified social deprivation and age to be associated with non‐attendance (with younger adults more likely to not attend than older adults), suggesting that our results may reflect broader trends and may be applicable to other childhood patient groups. The reasons that families do not attend outpatient clinic appointments has also been the subject of recent qualitative studies.^20^, ^21^ Common reasons reported included travel difficulties, competing priorities, and administrative issues (e.g. not receiving an appointment, difficulties in changing an appointment) that highlight the complexity that families face in balancing their child’s health needs and other priorities.^22^ These perspectives can help us reconsider non‐attendance as a weakness in the model of care provided through outpatient clinics, in that they rely on face‐to‐face contact between a patient and health care professional at a particular point in time. There is a need for increased acknowledgement that health care is not a ‘one‐size‐fits‐all’ provision and personalizing health care delivery should sit alongside the agenda to personalize therapeutics. Strategies such as mHealth (e.g. SMS reminders^23^) and telemedicine may help support this agenda. The increased use of telemedicine during the COVID‐19 pandemic has suggested that this is an acceptable alternative for many face‐to‐face consultations.^24^ Our results also underpin the need for improved coordination of care and integration of speciality care with a child’s local health care service and primary‐care team, to ensure all opportunities to optimize health and development are pursued. The increasing rate of non‐attendance that we identified makes these requirements time sensitive.

The strengths of our study include its size and the use of data from a CP register, which improved the precision of our study population and availability of clinical and sociodemographic descriptors, and the use of routinely collected administrative data. This is mandated by the NSW state government and ensured accurate estimation of attendance rates and service events. However, our methodology was not designed to explore the reasons that families did not attend, nor the reasons for the increase in non‐attendance over time. Other limitations of our methodology include missing pertinent factors in our available data, including residency status. Our data also did not allow us to identify appointments cancelled by families prior to their appointment (distinct from those cancelled by the hospital). As others have identified,^25^ this is another clinically important group, as they too represent a missed opportunity for health care.

Our study shows an association between non‐attendance at outpatient appointments and socioeconomic disadvantage, increasing age, recent non‐attendance, and cancelled or rescheduled appointments. These factors are readily identified and should be targeted when considering strategies to support families who may be experiencing difficulties with health care access. For example, clinicians can follow‐up families who have missed appointments and consider alternative ways of supporting children’s health where possible. Future studies to investigate barriers and facilitators for attendance to outpatient clinics and interventions to improve health care accessibility are warranted. This will enable the design and implementation of appropriate measures for uptake and access to care and services.

## Supporting information


**Figure S1:** Flow diagram of inclusions and exclusions in study.

**Table S1:** Proportions of children and likelihood of scheduling for major specialty outpatient clinics in children with cerebral palsy, 2012 to 2019.

**Table S2:** Proportions of children and likelihood of scheduling for major specialty outpatient clinics in children with cerebral palsy, 2012 to 2019.

**Table S3:** Rates and proportions of scheduled outpatient days by age group and major specialty outpatient clinics in children with cerebral palsy, 2012 to 2019.

## Data Availability

The data that support the findings of this study are available on request from the corresponding author. The data are not publicly available due to privacy or ethical restrictions.
