# Supplementary figures and images for: Non‐attendance at outpatient clinic appointments by children with cerebral palsy
Source: Dev Med Child Neurol. 2022 Mar 4;64(9):1106–13. doi: 10.1111/dmcn.15197 (PMC9545710; doi:10.1111/dmcn.15197)

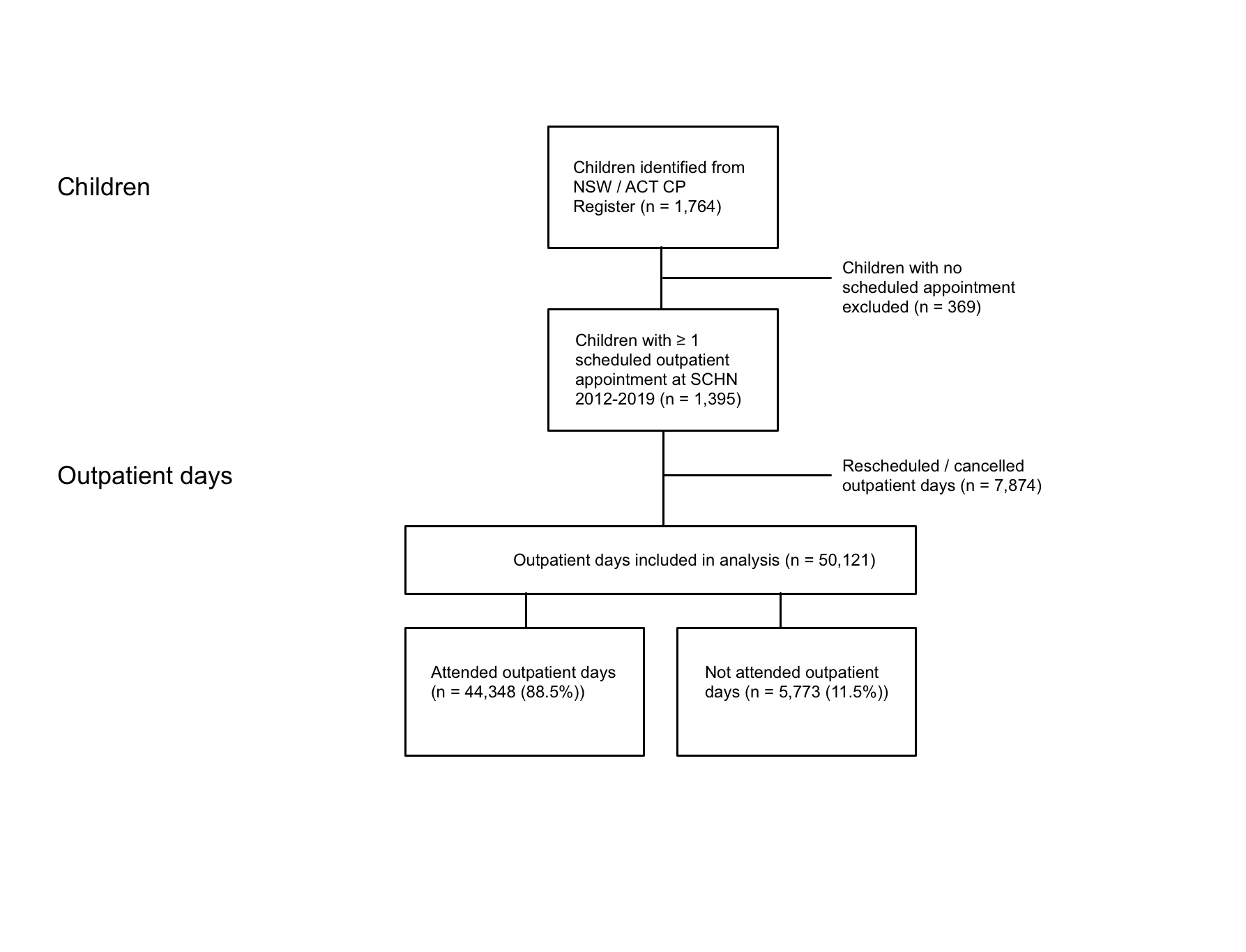

Supplement: Supplementary file 1 — Figure S1: Flow diagram of inclusions and exclusions in study. [file DMCN-64-1106-s004.jpg]
