# Supplementary material for: Non‐attendance at outpatient clinic appointments by children with cerebral palsy
Source: Dev Med Child Neurol. 2022 Mar 4;64(9):1106–13. doi: 10.1111/dmcn.15197 (PMC9545710; doi:10.1111/dmcn.15197)
Supplement: Supplementary file 2 — Table S1: Proportions of children and likelihood of scheduling for major specialty outpatient clinics in children with cerebral palsy, 2012 to 2019. [file DMCN-64-1106-s002.docx]

**Supplementary Table 1: List of medical and surgical specialties**

| Specialty Group | Specialties | | |
| --- | --- | --- | --- |
| Allied Health | Physiotherapy | | Social Work  Psychology |
|  | Occupational Therapy | |  |
|  | Speech Pathology | | Child life therapy |
|  | Dietetics | | Orthotics |
| General Medicine | General Medicine | | |
| Rehabilitation | Rehabilitation Medicine | | |
| Neurology / Neurosurgery | Neurology | Neurosurgery | |
| Other Medical Specialties | Adolescent Medicine | Neonatology | |
|  | Allergy & Immunology | Oncology | |
|  | Audiology | Pain | |
|  | Cardiology / Cardiothoracic | Palliative Care | |
|  | Developmental Medicine | Psychological Medicine | |
|  | Endocrinology | Renal Medicine | |
|  | Gastroenterology | Respiratory and Sleep Medicine | |
|  | Haematology | Rheumatology | |
|  | Infectious Diseases | Sports Medicine | |
|  | Metabolic medicine | Weight Management | |
| General Surgery | General Surgery | | |
| Orthopaedics | Orthopaedics | | |
| Other Surgical Specialties | Anaesthetics | Ophthalmology | |
|  | Craniofacial | Gynaecology | |
|  | Dental | Plastics | |
|  | Ears, Nose, Throat | Urology | |
|  | Cleft lip / palate | Burns | |
