# Supplementary material for: Non‐attendance at outpatient clinic appointments by children with cerebral palsy
Source: Dev Med Child Neurol. 2022 Mar 4;64(9):1106–13. doi: 10.1111/dmcn.15197 (PMC9545710; doi:10.1111/dmcn.15197)
Supplement: Supplementary file 3 — Table S2: Proportions of children and likelihood of scheduling for major specialty outpatient clinics in children with cerebral palsy, 2012 to 2019. [file DMCN-64-1106-s003.docx]

**Supplementary Table 2. Proportions of children and likelihood of scheduling for major specialty outpatient clinics in children with cerebral palsy, 2012-2019**

| Demographic and clinical factors | Scheduled appointments | Rehabilitation Medicine appointments | | Allied Health appointments | | Neurology appointments | | Orthopaedic appointments | |
| --- | --- | --- | --- | --- | --- | --- | --- | --- | --- |
|  | n (%) | n (%) | OR (95%CI) | n (%) | OR (95%CI) | n (%) | OR (95%CI) | n (%) | Odds Ratio |
| Total | 1395 (100) | 1147 (100) |  | 1100 (100) |  | 776 (100) |  | 604 (100) |  |
| Sex |  |  |  |  |  |  |  |  |  |
| Male | 831 (59.6) | 674 (58.8) | 0.83 (0.62-1.1) | 661 (60.1) | 0.83 (0.62-1.1) | 445 (57.3) | 0.81 (0.65-1.01) | 338 (56) | 0.77 (0.62-0.95) |
| Female | 564 (40.4) | 473 (41.2) | REF | 439 (39.9) | REF | 331 (42.7) | REF | 266 (44) | REF |
| Country of Birth |  |  |  |  |  |  |  |  |  |
| Australia | 1298 (93) | 1058 (92.2) | REF | 1027 (93.4) | REF | 727 (93.7) | REF | 561 (92.9) | REF |
| Overseas | 88 (6.3) | 83 (7.2) | 3.77 (1.51-9.39) | 71 (6.5) | 3.77 (1.51-9.39) | 46 (5.9) | 0.86 (0.56-1.33) | 42 (7) | 1.2 (0.78-1.85) |
| Preferred Language |  |  |  |  |  |  |  |  |  |
| English | 1214 (87) | 1005 (87.6) | REF | 964 (87.6) | REF | 672 (86.6) | REF | 538 (89.1) | REF |
| Other | 120 (8.6) | 97 (8.5) | 0.88 (0.54-1.42) | 94 (8.5) | 0.88 (0.54-1.42) | 77 (9.9) | 1.44 (0.98-2.13) | 61 (10.1) | 1.3 (0.89-1.89) |
| Remoteness |  |  |  |  |  |  |  |  |  |
| Major cities | 941 (67.5) | 784 (68.4) | REF | 748 (68) | REF | 539 (69.5) | REF | 539 (89.2) | REF |
| Regional / Remote | 448 (32.1) | 357 (31.1) | 0.79 (0.59-1.05) | 346 (31.5) | 0.79 (0.59-1.05) | 233 (30) | 0.81 (0.64-1.01) | 233 (38.6) | 0.9 (0.71-1.13) |
| IRD Quintile |  |  |  |  |  |  |  |  |  |
| 1 (most disadvantaged) | 274 (19.6) | 218 (19) | 0.57 (0.37-0.89) | 212 (19.3) | 0.57 (0.37-0.89) | 164 (21.1) | 1.22 (0.88-1.69) | 124 (20.5) | 1.05 (0.76-1.44) |
| 2 | 210 (15.1) | 165 (14.4) | 0.54 (0.34-0.86) | 154 (14) | 0.54 (0.34-0.86) | 106 (13.7) | 0.83 (0.59-1.18) | 89 (14.7) | 0.93 (0.66-1.32) |
| 3 | 275 (19.7) | 216 (18.8) | 0.54 (0.35-0.83) | 205 (18.6) | 0.54 (0.35-0.83) | 146 (18.8) | 0.93 (0.67-1.27) | 105 (17.4) | 0.78 (0.57-1.08) |
| 4 | 288 (20.6) | 244 (21.3) | 0.82 (0.52-1.29) | 236 (21.5) | 0.82 (0.52-1.29) | 168 (21.6) | 1.15 (0.84-1.57) | 130 (21.5) | 1.04 (0.76-1.43) |
| 5 (least disadvantaged) | 342 (24.5) | 298 (26) | REF | 287 (26.1) | REF | 188 (24.2) | REF | 151 (25) | REF |
| GMFCS |  |  |  |  |  |  |  |  |  |
| I-III | 998 (71.5) | 819 (71.4) | REF | 756 (68.7) | REF | 492 (63.4) | REF | 360 (59.6) | REF |
| IV-V | 342 (24.5) | 309 (26.9) | 2.05 (1.38-3.03) | 312 (28.4) | 2.05 (1.38-3.03) | 248 (32) | 2.71 (2.08-3.55) | 231 (38.2) | 3.69 (2.84-4.79) |
| Predominant motor type |  |  |  |  |  |  |  |  |  |
| Spastic | 1010 (72.4) | 865 (75.4) | REF | 815 (74.1) | REF | 502 (64.7) | REF | 457 (75.7) | REF |
| Dyskinetic | 192 (13.8) | 168 (14.6) | 1.17 (0.74-1.86) | 164 (14.9) | 1.17 (0.74-1.86) | 135 (17.4) | 2.4 (1.72-3.34) | 86 (14.2) | 0.98 (0.72-1.34) |
| Other | 171 (12.3) | 110 (9.6) | 0.3 (0.21-0.43) | 109 (9.9) | 0.3 (0.21-0.43) | 123 (15.9) | 2.59 (1.82-3.7) | 56 (9.3) | 0.59 (0.42-0.83) |
| Intellectual Disability |  |  |  |  |  |  |  |  |  |
| Yes | 645 (46.2) | 526 (45.9) | 0.66 (0.48-0.91) | 527 (37.8) | 0.66 (0.48-0.91) | 433 (67.8) | 3.06 (2.41-3.9) | 331 (62.7) | 1.7 (1.34-2.15) |
| No | 515 (36.9) | 448 (39.1) | REF | 404 (29) | REF | 206 (32.2) | REF | 197 (37.3) | REF |
| Not reported | 235 (16.8) | 235 (0) |  | 235 (0) |  | (0) |  | (0) |  |
| Epilepsy |  |  |  |  |  |  |  |  |  |
| Yes | 394 (28.2) | 323 (32.3) | 0.79 (0.57-1.09) | 326 (34.1) | 0.79 (0.57-1.09) | 325 (48.8) | 6.28 (4.68-8.44) | 208 (39.6) | 1.69 (1.32-2.16) |
| None or resolved | 796 (57.1) | 678 (67.7) | REF | 631 (65.9) | REF | 341 (51.2) | REF | 317 (60.4) | REF |
| Not reported | 205 (14.7) | 205 (0) |  | (0) |  | (0) |  | (0) |  |
