# Supplementary material for: Non‐attendance at outpatient clinic appointments by children with cerebral palsy
Source: Dev Med Child Neurol. 2022 Mar 4;64(9):1106–13. doi: 10.1111/dmcn.15197 (PMC9545710; doi:10.1111/dmcn.15197)
Supplement: Supplementary file 4 — Table S3: Rates and proportions of scheduled outpatient days by age group and major specialty outpatient clinics in children with cerebral palsy, 2012 to 2019. [file DMCN-64-1106-s001.docx]

**Supplementary Table 3. Rates and proportions of scheduled outpatient days by age group and major specialty outpatient clinics in children with cerebral palsy, 2012-2019**

| Specialty Group n (%) | Total outpatient days (n) | Total outpatient days by age group n (%) | | | p |
| --- | --- | --- | --- | --- | --- |
|  |  | 0 to 4 years | 5 to 9 years | 10 to 14 years |  |
| Total outpatient days | 50121 | 20204 (40) | 22242 (44) | 7675 (15) | <0.0001 |
| Specialty Type | | | | | |
| Allied Health | 19008 | 7104 (37) | 9023 (47) | 2881 (15) | <0.0001 |
| General Medicine | 1863 | 813 (44) | 822 (44) | 228 (12) | 0.0002 |
| Rehabilitation Medicine | 14918 | 4874 (33) | 7397 (50) | 2647 (18) | <0.0001 |
| Neurology | 4982 | 2707 (54) | 1741 (35) | 534 (11) | <0.0001 |
| General Surgery | 1030 | 552 (54) | 364 (35) | 114 (11) | <0.0001 |
| Orthopaedics | 4433 | 804 (18) | 2306 (52) | 1323 (30) | <0.0001 |
